# Dosimetric comparison and biological evaluation of fixed-jaw intensity-modulated radiation therapy for T-shaped esophageal cancer

**DOI:** 10.1186/s13014-021-01882-7

**Published:** 2021-08-19

**Authors:** Hua Chen, Ying Huang, Hao Wang, Yan Shao, Ning J. Yue, Hengle Gu, Yanhua Duan, Aihui Feng, Zhiyong Xu

**Affiliations:** 1 Department of Radiation Oncology, Shanghai Chest Hospital, Shanghai Jiao Tong University, No. 241 West Huaihai Road, Xuhui District, Shanghai, 200030 China; 2 Department of Radiation Oncology, Rutgers Cancer Institute of New Jersey, Rutgers University, New Brunswick, NJ 08903 USA

**Keywords:** T-shaped esophageal cancer, Fixed-jaw, Jaw tracking, Static jaw, VMAT, Radiation pneumonitis

## Abstract

**Background:**

To evaluate the dosimetric and biological benefits of the fixed-jaw (FJ) intensity-modulated radiation therapy (IMRT) technique for patients with T-shaped esophageal cancer.

**Methods:**

FJ IMRT plans were generated for thirty-five patients and compared with jaw tracking (JT) IMRT, static jaw (SJ) IMRT and JT volumetric modulated arc therapy (VMAT). Dosimetric parameters, tumor control probability (TCP) and normal tissue complication probability (NTCP), monitor units (MUs), delivery time and gamma passing rate, as a measure of dosimetric verification, were compared. The correlation between the length of PTV-C below the upper boundary of lung tissue (PTV-C_inferior_) and dosimetric parameters and NTCP of the lung tissue were analyzed.

**Results:**

The homogeneity and conformity of the target in the four plans were basically equivalent. When compared to the JT IMRT and SJ IMRT plans, FJ IMRT plan led to a statistically significant improvement in the NTCP and low-middle dosimetric parameters of the lung, and the improvement had a moderately positive correlation with the length of PTV-C_inferior_, with a correlation coefficient ranging from 0.523 to 0.797; the FJ IMRT plan exhibited better lung sparing in low-dose volumes than the JT VMAT plan. The FJ IMRT plan had similar MUs (888 ± 99) and delivery times (516.1 ± 54.7 s) as the JT IMRT plan (937 ± 194, 522 ± 5.6 s) but higher than SJ IMRT (713 ± 137, 488.8 ± 45.2 s) and JT VMAT plan (517 ± 59, 263.7 ± 43.3 s).

**Conclusions:**

The FJ IMRT technique is superior in reducing the low-dose volumes of lung tissues for patients with T-shaped esophageal cancer.

## Background

Esophageal cancer is one of the most common malignant diseases worldwide [1]. Prophylactic irradiation of the lymph nodes in the bilateral supraclavicular region and upper-middle mediastinal region is a routine method for radical radiotherapy or postoperative radiotherapy for patients with esophageal cancer. The target volume of this type of treatment is usually large and encompasses a T-shape from a postero-anterior view, so radiotherapy might deliver a substantial dose to the lung, heart, and spinal cord. Radiation pneumonitis (RP) is the most common dose-limiting toxicity of thoracic radiotherapy, and it occurs in 5–50% of patients [2, 3]. RP induces emphysema, pulmonary fibrosis, and inflammation [4, 5], which can seriously affect the quality of life of patients and pose life-threatening risks. Previous studies have shown that the incidence of RP depends on the volume of and dose to the irradiated lung [4, 5]. Therefore, it is desirable to minimize radiation to the lung in the treatment of esophageal cancer patients.

At present, the static jaw intensity-modulated radiation therapy (IMRT) technique, jaw tracking IMRT technique, and volumetric modulated arc therapy (VMAT) technique are mainly used in the clinical treatment and research of esophageal cancer. In the conventional static jaw IMRT technique, the leaves of the multi-leaf collimators (MLCs) continuously move at various speeds during irradiation, while the upper jaw and lower jaw remain stationary [6]. For most modern linear accelerators, the collimator jaw positions can be set to automatically track the shapes of the subfields in IMRT treatment. The collimator jaws move automatically to be as close as possible to the MLC apertures during dose delivery to further reduce radiation leakage and transmission [7]. With dynamic MLC motion and variable gantry speed modulation and dose rate, the VMAT technique was developed as a novel form of IMRT [8]. VMAT could not only produce similar or better dose distributions than IMRT but also achieved a reduction in monitor units (MUs) [9–12]. Feng et al. [6] compared the jaw tracking IMRT technique and static jaw IMRT technique for various common disease sites, such as the esophagus, lung, head & neck, abdomen and pelvis, and found that the jaw tracking IMRT technique can better protect normal tissues than the static jaw IMRT technique. Several studies have reported that full-arc VMAT technology significantly reduced the V_20_ of the lungs compared with the jaw tracking IMRT technique, but full-arc VMAT also increased the low-dose volumes of the lungs, such as V_5_ and V_13,_ for upper and middle thoracic esophageal cancer [8, 13], where V_x_ was the volume that was irradiated above a designated dose of x Gy.

Recently, a fixed-jaw IMRT technique has been proposed. The position of the collimator jaws in fixed-jaw IMRT can be adjusted according to the location, size and shape of the planning target volume (PTV) and organs at risk (OARs) [14–17]. This technique is often used to optimize the irradiation of large fields by locking the collimator’s jaws in a fixed position so to avoid split-fields and to further protect the critical OARs. Chen et al. [14] showed that in the radiation treatment of ovarian cancer, compared with the traditional static jaw IMRT plan, the fixed-jaw IMRT plan could further reduce the dose to the ovaries for ovarian cancer patients and generate plans with lower monitor units (MUs). Wang et al. [16] compared a fixed-jaw IMRT plan with a jaw tracking IMRT plan, which were used during radiotherapy for peripheral lung cancer with mediastinal lymph node metastasis. The results showed that the fixed-jaw IMRT technique could further reduce the dose to normal lung tissues. Fixed-jaw IMRT has also been indicated in breast cancer to effectively reduce the low dose to normal tissues while maintaining target coverage [17]. Song et al. also showed the advantages of improving the quality of the IMRT plan with the use of the fixed-jaw technique for cervical and upper thoracic esophageal cancer [18].

However, due to the dose-limiting adjacent critical structures and large interpatient variability in the width and depth of T-shaped esophageal cancer, radiotherapy treatment planning for these tumors is relatively challenging. Conventional radiotherapy techniques have difficulty meeting the dosimetric constraints, especially regarding lung sparing for T-shaped esophageal cancer with long targets in the superior-inferior direction. This is mainly because the T-shaped target volumes include the wide bilateral supraclavicular lymph nodes, and the extension of the target below the upper boundary of lung tissue is long. The posterior gantry angles in conventional IMRT techniques need to be employed to maintain target conformity, leading to irradiation of a large lung volume and a potential increase in the incidence of RP. The low-dose volumes of the lungs may be increased when the full-arc VMAT technique is used [8, 19].

In our previous study [20], we compared the dosimetric effects of the partial arc VMAT, full arc VMAT and IMRT techniques on upper esophageal cancer, which were all combined with jaw tracking, and found that compared with the IMRT technique, the full arc VMAT technique significantly increased the V_5_ of the total lung, but the partial arc VMAT technique did not increase the V_5_ value and could also significantly reduce the high-dose volumes of the total lung, such as the V_20_ and V_30_. As an alternative to VMAT, it can be of interest to investigate whether applying IMRT with FJ rather than SJ can improve the dosimetric results and potential biological effects for T-shaped esophageal cancers. Moreover, it is also unclear whether the efficacy of sparing OARs (especially the lungs) with the fixed-jaw IMRT technique correlates with the extension of the target below the upper boundary of lung tissue. Indeed, although the target width of the bilateral supraclavicular lymph nodes in T-shaped esophageal cancer is similar among patients, the length of the target in the inferior direction can be quite different.

This study aimed to explore the feasibility of the fixed-jaw IMRT technique for T-shaped esophageal cancer in comparison with the jaw tracking IMRT, static jaw IMRT and jaw tracking VMAT techniques in terms of dosimetry parameters, TCP and NTCP values. To quantify the benefit of lung sparing with the fixed-jaw IMRT technique, the correlations between the length of the target below the upper boundary of lung tissue and the amount of differences in dosimetry parameters and NTCP of the lungs in FJ IMRT relative to the other three techniques were also analyzed.

## Methods

### Patient characteristics

Thirty-five patients with histologically or cytologically confirmed T-shaped esophageal cancer with different lengths of target below the upper boundary of lung tissue who were treated with radiotherapy between January 2017 and May 2018 were selected for this study. The detailed patient characteristics are presented in Table 1. For each patient, the radiotherapy CT simulation was conducted on a Siemens Somatom Definition AS CT Scanner System (Siemens Healthcare, Erlangen, Germany). The patient laid in the supine position, with both arms placed at the sides of body, and was immobilized with a head and upper thoracic thermoplastic mask. The patient was scanned from cervical vertebrae C3 to the lower edge of the liver, including the entire lung, with a slice thickness of 5 mm. Treatment planning was performed on a Pinnacle v9.10 treatment planning system (Philips Healthy, Fitchburg, WI).

**Table 1:** Patient characteristics

Characteristics	Value
*Age *(*year*)	
Median	62
Range	46–75
*Gender *(*n*)	
Male	28
Female	7
*T stage *(*n*)	
T2	8
T3	27
*N stage *(*n*)	
N0	11
N1-3	24
*Length of PTV* (*cm*)	
Mean	18. ± 2.3
Range	10.5–24
*Length of PTV-C*_*inferior*_ (*cm*)	
Mean	12.1 ± 2.5
Range	4.5–19

PTV, Planning target volume; PTV-C_inferior_, the part of the PTV-C below the upper boundary of lung tissue

### Contouring the targets and OARs

The target volumes and OARs of each patient were delineated by a qualified physician with more than 10 years of experience in clinical radiation oncology. The gross tumor volume (GTV) was the esophageal cancer lesion delineated on the CT images with the aid of the esophagogram, esophagoscopy images and pathology report. The clinical target volume (CTV) was defined as the GTV plus the bilateral supraclavicular lymph nodes and upper-middle mediastinal lymph nodes. The planning target volume (PTV) was expanded from GTV in this study, to account for the uncertainties of the setup error, respiratory movement, heartbeat, esophageal peristalsis and movement.

PTV-G was defined by expanding 6 mm isotropically from the GTV, and PTV-C isotropically expanded the CTV by a 6–8-mm margin, excluding any volumes that extended beyond the skin. The OARs included the total lung, spinal cord and heart. Total lung was defined as the lung volume minus the GTV.

### Treatment planning

Each patient had four plans: the jaw tracking (JT) IMRT plan, the static-jaw (SJ) IMRT plan, the fixed-jaw (FJ) IMRT plan and the JT VMAT plan. All plans were created on the Pinnacle v9.10 treatment planning system with a 6 MV photon beam from an Edge™ linear accelerator (Varian Medical Systems, Palo Alto, CA). The four plans for each patient used the same isocenter. For all plans, a simultaneous integrated boost technique was applied: a total of 50.4 Gy in 28 fractions was prescribed to the PTV-C, and a total of 60.2 Gy in 28 fractions was prescribed to the PTV-G. The optimization goals were to deliver the 60.2 Gy to at least 95% of the PTV-G and 95% of the 50.4 Gy to at least 99% of the PTV-C. The constraints to the OARs were shown in Table 2.

**Table 2:** The constraints to the OARs

OARs	Objective
Total lung	V_5_ ≤ 65%
	V_20_ ≤ 25%
	V_30_ ≤ 20%
	MLD ≤ 15 Gy
Spinal cord	D_max_ ≤ 45 Gy
Heart	D_mean_ ≤ 26 Gy
	V_30_ ≤ 40%
	V_40_ ≤ 30%

OAR, organ at risk; V_x_, the relative volume of an OAR receives a dose of at least x Gy; MLD, mean lung dose; D_max_, maximum dose; D_mean_, mean dose

**Fig. 1:**
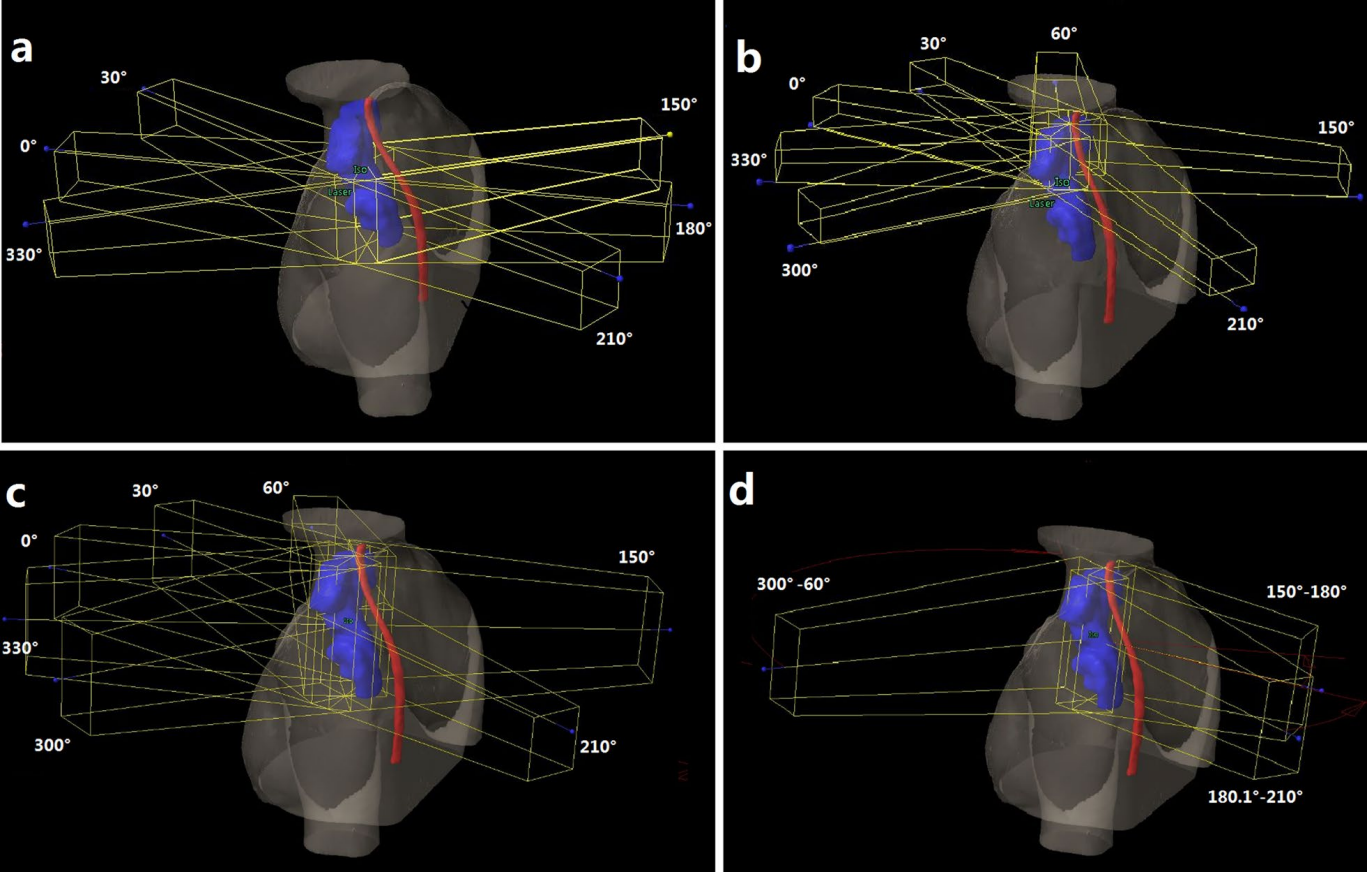
**a** and **b** Beam arrangement for the FJ IMRT plan. **c** Beam arrangement for the JT IMRT and SJ IMRT plan. **d** Beam arrangement for the JT VMAT plan. PTV-C (blue), body (antiquewhite), spinal cord (red). JT IMRT: jaw-tracking intensity-modulated radiation therapy; SJ IMRT: static jaw intensity-modulated radiation therapy; JT VMAT: jaw-tracking volumetric modulated arc therapy; FJ IMRT: fixed-jaw intensity-modulated radiation therapy

**Fig. 2:**
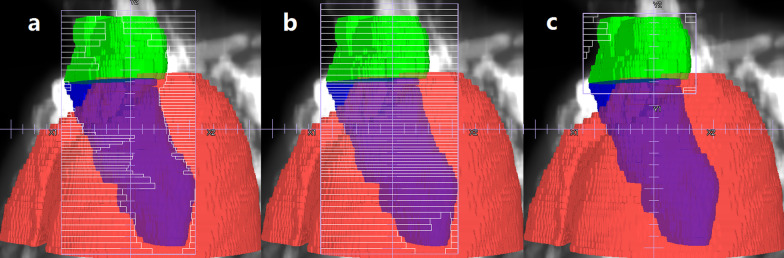
The field jaw positions with an angle of 60° for **a** JT IMRT plan, **b** SJ IMRT plan and **c** FJ IMRT plan. PTV-C_superior_: green; PTV-C_inferior_: blue; lung: tomato. JT IMRT: jaw-tracking intensity-modulated radiation therapy; SJ IMRT: static jaw intensity-modulated radiation therapy; JT VMAT: jaw-tracking volumetric modulated arc therapy; FJ IMRT: fixed-jaw intensity-modulated radiation therapy

For the JT IMRT plan, based on our clinical experiences, as shown in Fig. 1c, seven beams with gantry angles of 210°, 300°, 330°, 0°, 30°, 60° and 150° were used. Due to the MLC limitations of the Edge linear accelerator, the collimator was set to 90° if the treatment field was longer than 22 cm. The jaw tracking function on Pinnacle was selected, and the plans automatically set the positions of each pair of jaws to reduce radiation leakage and transmission. Thus, during the delivery of each beam, the collimator jaws moved automatically to be as close as possible to the MLC apertures (Fig. 2a).

For the SJ IMRT plan, beam parameter settings identical to the corresponding JT IMRT plans were employed (Fig. 1c), except that the jaw tracking function on the planning treatment planning system was not selected; thus, the collimator jaws did not move during the delivery of each beam (Fig. 2b).

Based on previous research [20], the JT VMAT plan used a partial arc with jaw tracking technology and was designed as three partial arcs: 180.1°–210° (CW, CCW), 300°–60° (CW, CCW), and 150°–180° (CW, CCW); the collimator angle for all partial arcs was 45° (Fig. 1d).

In the FJ IMRT plan, to minimize the transmitted dose to the lung tissue as much as possible, with the upper boundary of lung tissue as the dividing boundary, the T-shaped PTV-C was divided into two parts in the superior-inferior direction, PTV-C_superior_ and PTV-C_inferior_. The gantry angles for FJ IMRT plan were adjusted slightly based on the JT plan. As the PTV-C_superior_ was wide in the left–right direction, to reduce the dose to the spinal cord and ensure conformity, as shown in Fig. 1b, seven beams were used, with gantry angles of 210°, 300°, 330°, 0°, 30°, 60°and 150° (Fig. 2c), and the collimator angle was set to 90° if the MLC cannot cover the target volume when collimator angle was 0°. The jaw position was fixed to cover PTV-C_superior_. For the PTV-C_inferior_, as shown in Fig. 1a, 4–6 beams with gantry angles of 210°, 330°, 0°, 30°, 150° or 180° were used mainly to reduce the exposure of normal tissues surrounding the PTV-C_inferior_, especially lungs and spinal cord. Three different cases/scenarios were possible: (1) if the PTV-C_inferior_ was close to the posterior chest wall in anterior–posterior direction, 4 beams could be used, i.e., with gantry angles of 210°, 0°, 150° and 180°, respectively; (2) if the PTV-C_inferior_ was close to the anterior chest wall in anterior–posterior direction, 4 beams could be used also, but with gantry angles of 330°, 0°, 30° and 180°, respectively; (3) if one part of the PTV-C_inferior_ was close to the anterior chest wall, and another part was relatively close to posterior chest wall, then 5–6 beams could be used, with gantry angles of 330°, 0°, 30°, 150°, 210° and/or 180°, respectively. The lower collimator jaws were fixed to cover PTV-C_inferior_. To ensure that there won’t be hot or cold spots in case of setup errors, the overlapping width of the two jaw openings in the superior-inferior direction was 1.0–2.0 cm.

The Auto-Planning module of the Pinnacle treatment planning system was used for dose optimization. The optimization parameters were kept the same for the four plans. Dose optimization was performed with direct machine parameter optimization and calculated with the collapsed cone convolution superposition algorithm and a 2.5 mm dose grid.

### Dosimetric evaluation parameters

A dose-volume histogram (DVH) was used to assess the dose distributions in the target volume and OARs. The target evaluation parameters included D_2_, D_98_, target conformity index (CI) [21], and target homogeneity index (HI) [22], where D_x_ was the minimum dose to the hottest x% of the PTV.

The CI was defined as follows:

$${\text{CI}} = \frac{{{\text{V}}_{{{\text{T}},{\text{ref}}}}^{2} }}{{{\text{V}}_{{\text{T}}} \times {\text{V}}_{{{\text{ref}}}} }}$$

where V_T,ref_ is the PTV receiving the prescribed dose, V_T_ is the PTV volume, and V_ref_ is the volume of all regions receiving the prescribed dose. A CI value closer to 1 indicates better target dose conformity.

HI was defined as follows:

$${\text{HI}} = \frac{{{\text{D}}_{2} - {\text{D}}_{98} }}{{{\text{D}}_{{\text{p}}} }}$$

where D_2_ is the corresponding dose for 2% of the target volume on the cumulative DVH, D_98_ is the corresponding dose for 98% of the target volume, and D_p_ is the prescription dose. A lower HI value indicates a more homogenous dose distribution.

The parameters used to evaluate the OARs sparing included MLD, V_5_, V_10_, V_13_, V_15_, V_20_ and V_30_ of the total lung, D_max_ of the spinal cord, the mean dose, and V_30_ and V_40_ of the heart. There were five patients whose targets were located in the cervical and upper thoracic esophageal regions, and the V_30_ and V_40_ of the heart were almost 0, so the heart data of thirty patients were compared in this study.

### TCP and NTCP calculation

The tumor control probability (TCP) of the target and normal tissue complication probability (NTCP) of total lung, spinal cord and heart were used to evaluate the treatment plans. Both the TCP and NTCP calculations were performed using programs developed in-house with MATLAB R2019a (The MathWorks Inc., MA, USA). The TCP calculation was based on the equivalent uniform dose (EUD) model [23]. The equations were as follows:

$${\text{TCP}} = \frac{1}{{1 + \left( {\frac{{{\text{TCD}}_{50} }}{{{\text{EUD}}}}} \right)^{{4\gamma_{50} }} }}$$

$${\text{EUD}} = \left[ {\mathop \sum \limits_{{{\text{i}} = 1}} {\text{v}}_{{\text{i}}} *{\text{D}}_{{\text{i}}}^{{\text{a}}} } \right]^{{\frac{1}{{\text{a}}}}}$$

where TCD_50_ (i.e., the tumor dose required to produce a 50% TCP), ${\gamma }_{50}$ (i.e., the slope of dose response at 50% TCP) and $\mathrm{a}$ (i.e., a unitless parameter which describes the magnitude of the volume effect to tumor or the normal structure) were 51.24 Gy, 0.83 and 0.30, respectively. v_i_ is the relative volume related to dose voxel D_i_ [24].

The NTCP calculation was based on the Lyman–Kutcher–Burman model [25]. The equations were as follows:

$${\text{NTCP}} = \frac{1}{{\sqrt {2{\uppi }} }}\mathop \smallint \limits_{ - \infty }^{{\text{t}}} {\text{e}}^{{ - \frac{{{\text{x}}^{2} }}{2}}} {\text{dx}}$$

$${\text{t}} = \frac{{{\text{D}}_{{{\text{eff}}}} - {\text{TD}}_{50} }}{{{\text{m}}*{\text{TD}}_{50} }}$$

$${\text{D}}_{{{\text{eff}}}} = \left( {\mathop \sum \limits_{{\text{i}}} {\text{v}}_{{\text{i}}} *{\text{D}}_{{\text{i}}}^{{\text{n}}} } \right)^{{\frac{1}{{\text{n}}}}}$$

where TD_50_ represents the dose to the whole organ (or reference volume) which would lead to a complication probability of 50 percent. m is a measure of the slope of the sigmoid curve represented by the integral of the normal distribution. n is a parameter which describes the magnitude of the volume effect. v_i_ is the relative volume related to dose voxel D_i_. For pneumonitis, the TD_50_, n and m published by Semenko [26] were 29.9 Gy, 1 and 0.41, respectively. For myelitis/necrosis, the parameters published by Burman [27] were adopted, with TD_50_ = 66.5 Gy, n = 0.05, and m = 0.175. For pericarditis, the parameters published by Burman [27] were also adopted, with TD_50_ = 48.0 Gy, n = 0.35, m = 0.10.

The voxel-based fractionation correction was performed according to the EQD_2_ formula prior to calculation TCP and NTCP [28]. The EQD_2_ was the biologically equivalent physical dose given in 2 Gy per fraction of partial volume v_i_. The equation was as follows:

$${\text{EQD}}_{2} = {\text{D}}_{{\text{i}}} \left( {1 + \frac{{{\text{d}}_{{\text{i}}} }}{\alpha /\beta }} \right)$$

where ${\text{d}}_{{\text{i}}}$ is the dose per fraction size of the treatment course. $\alpha /\beta$ is the tissue-specific LQ parameters of the organ being exposed. In this study, $\alpha /\beta$ was set to 3 Gy for lung, $\mathrm{\alpha }/\upbeta$ was set to 2 Gy for spinal cord.

### Correlation analyses

To investigate whether the FJ IMRT plan can protect the OARs better than the other three plans by increasing the length of PTV-C_inferior_, the correlations between length of PTV-C_inferior_ and the amount of difference in dosimetric parameters and NTCP of lungs in FJ IMRT technique relative to the other three techniques were calculated. For example, the difference in V_5_ of the total lung between the FJ IMRT plan and JT IMRT plan was defined as ΔV_5 JT-FJ_. Similarly, the difference in V_5_ for the total lung between the FJ IMRT plan and SJ IMRT plan was defined as ΔV_5 SJ-FJ_, and the difference in V_5_ for the total lung between the FJ IMRT plan and JT VMAT plan was defined as ΔV_5 VMAT-FJ_. The evaluation parameters for the total lung included the MLD, V_5_, V_10_, V_13_, V_15_, V_20_ and V_30_ and NTCP.

### Total MU, delivery time and plan verification

In addition to the dosimetric quality, the FJ IMRT and other three techniques were compared in terms of total MUs and delivery time.

To verify that the JT IMRT, SJ IMRT, FJ IMRT and JT VMAT plans could be reliably delivered, dosimetric verification was performed and evaluated in terms of the γ passing rate (3%, 3 mm, local approach, threshold: 5%). The Pinnacle treatment planning system was used to create plans for verification by recalculating each treatment field of plans to an OCTAVIUS 4D phantom (PTW, Freiburg, Germany) containing a 2D detector arrays (PTW, Freiburg, Germany). The measured dose planes were compared with the computed dose distribution using VeriSoft® software (PTW-Freiburg, Freiburg, Germany).

Delivery time was defined as the time from when the beam was turned on for the first field to when the beam was turned off after the last field while the plans were delivered to the phantom.

### Statistical analysis

All the results are reported as the mean ± standard deviation. The statistical analysis was performed using SPSS Statistics v22.0 software (IBM Corp., Armonk, NY, USA). A one-way ANOVA with LSD post hoc t-test was performed to analyze the differences between four plans. Pearson correlation tests were performed to evaluate the correlations between length of PTV-C_inferior_ and the amount of difference in dosimetric parameters and NTCP of lungs in FJ IMRT plan relative to the other three plans. A *p* value < 0.05 was considered statistically significant.

## Results

**Fig. 3:**
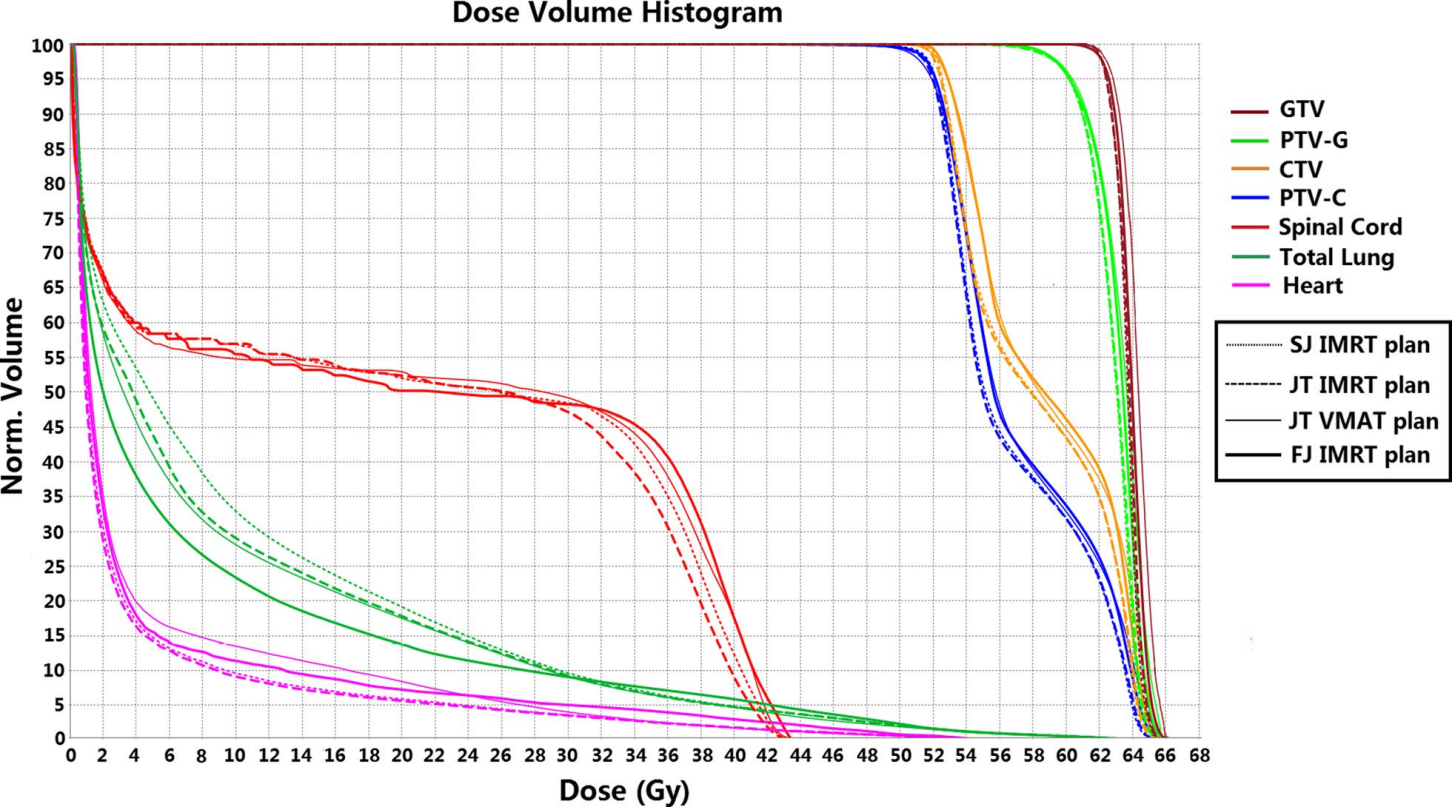
The comparison of DVHs for the PTV and OARs of the JT IMRT, SJ IMRT, JT VMAT and FJ IMRT plans for patient 1. DVH: dose-volume histogram; PTV: planning target volume; GTV: gross tumor volume; CTV: clinical target volume; OAR: organ at risk; JT IMRT: jaw-tracking intensity-modulated radiation therapy; SJ IMRT: static jaw intensity-modulated radiation therapy; JT VMAT: jaw-tracking volumetric modulated arc therapy; FJ IMRT: fixed-jaw intensity-modulated radiation therapy

One hundred forty plans were generated for the thirty-five patients, and all the plans met the preset clinical dose limit. Table 3 presents the comparison of the PTV dosimetric parameters of the four techniques. Figure 3 displays the DVH comparison for a representative patient. As shown in Table 3, the D_2_, D_98_, CI and HI of PTV-G and D_98_ of PTV-C in the FJ IMRT plan were not significantly different from those of the JT IMRT, SJ IMRT and JT VMAT plans. Therefore, four techniques obtained plans of comparable quality in terms of target coverage, conformity and homogeneity, always satisfying the clinical goals.

**Table 3:** Comparisons of dosimetric parameters of PTV

	JT IMRT	SJ IMRT	JT VMAT	FJ IMRT	ANOVA *p* value	Post hoc *p* value		
						FJ IMRT versus JT IMRT	FJ IMRT versus SJ IMRT	FJ IMRT versus JT VMAT
*PTV-G*								
D_2_ (Gy)	63.65 ± 0.73	63.62 ± 0.75	63.79 ± 0.80	63.67 ± 0.66	0.800	0.459	0.347	0.504
D_98_ (Gy)	59.43 ± 0.38	59.47 ± 0.37	59.45 ± 0.34	59.42 ± 0.33	0.930	0.775	0.864	0.665
CI	0.84 ± 0.03	0.84 ± 0.03	0.84 ± 0.05	0.84 ± 0.03	0.889	0.846	0.599	0.912
HI	0.07 ± 0.02	0.07 ± 0.02	0.08 ± 0.02	0.07 ± 0.01	0.846	0.827	0.561	0.771
*PTV-C*								
D_2_ (Gy)	62.89 ± 0.80	62.83 ± 0.81	63.78 ± 0.86	63.80 ± 0.88	< 0.001	< 0.001	< 0.001	0.922
D_98_ (Gy)	50.61 ± 0.73	50.60 ± 0.75	50.58 ± 0.80	50.62 ± 0.65	0.998	0.962	0.951	0.854

All values are shown as the mean ± standard deviation

PTV, planning target volume; JT IMRT, jaw-tracking intensity-modulated radiation therapy; SJ IMRT, static jaw intensity-modulated radiation therapy; JT VMAT, jaw-tracking volumetric modulated arc therapy; FJ IMRT, fixed-jaw intensity-modulated radiation therapy; D_x_, the minimum dose to the hottest x% of the PTV; CI, conformity index; HI, homogeneity index

Table 4 summarizes the dosimetric comparisons for the OARs in all patients. The DVHs of the OARs of a representative patient are also shown in Fig. 3. As shown in Table 4, the FJ IMRT plan presented advantages in reducing the volumes of the total lung receiving different dose levels. For the MLD, V_5_, V_10_, V_13_, V_15_ and V_20_ of the total lung, the values in the FJ IMRT plan were significantly lower than those in the JT IMRT and SJ IMRT plans. Compared with the JT VMAT plan, the FJ IMRT plan significantly reduced the V_5_, V_10_ and V_13_ of total lung, but not the MLD, V_13_, V_15_ and V_20_. Comparing with JT IMRT plan, the FJ IMRT plan reduced V5 by 7.5%. Similarly, Comparing with SJ IMRT and JT VMAT plan, the FJ IMRT plan reduced V5 by 10.0% and 5.4%, respectively. The V_30_ of the total lung, the D_max_ of the spinal cord, and the D_mean_, V_30_ and V_40_ of the heart were below the clinically acceptable tolerance, and there was no significant difference between the FJ IMRT plan and the other three plans.

**Table 4:** Comparisons of dosimetric parameters of OARs

OARs	JT IMRT	SJ IMRT	JT VMAT	FJ IMRT	ANOVA *p* value	Post hoc *p* value		
						FJ IMRT versus JT IMRT	FJ IMRT versus SJ IMRT	FJ IMRT versus JT VMAT
*Total lung*								
MLD (Gy)	9.49 ± 2.10	9.98 ± 2.25	8.93 ± 1.89	8.46 ± 1.95	0.016	0.040	0.003	0.335
V_5_ (%)	43.32 ± 11.74	45.80 ± 12.38	41.18 ± 11.37	35.80 ± 10.16	0.003	0.007	< 0.001	0.005
V_10_ (%)	32.35 ± 8.72	33.30 ± 9.50	30.16 ± 6.61	25.03 ± 7.53	< 0.001	< 0.001	< 0.001	0.001
V_13_ (%)	27.03 ± 7.13	28.52 ± 7.75	25.82 ± 5.22	21.98 ± 5.94	< 0.001	0.002	< 0.001	0.016
V_15_ (%)	24.51 ± 6.12	25.84 ± 6.63	21.24 ± 4.56	19.89 ± 5.22	< 0.001	0.001	< 0.001	0.325
V_20_ (%)	18.31 ± 3.95	19.32 ± 4.15	17.09 ± 3.58	16.14 ± 3.96	0.005	0.022	0.001	0.310
V_30_ (%)	8.95 ± 2.62	9.59 ± 2.67	9.01 ± 2.60	8.96 ± 2.28	0.680	0.990	0.307	0.932
*Spinal cord*								
D_max_ (Gy)	42.98 ± 1.07	43.13 ± 1.05	43.10 ± 1.12	43.11 ± 0.99	0.991	0.794	0.971	0.982
*Heart*								
D_mean_ (Gy)	8.90 ± 7.44	8.98 ± 8.35	9.07 ± 7.44	9.02 ± 7.64	0.998	0.948	0.981	0.979
V_30_ (%)	14.39 ± 10.64	14.31 ± 9.47	14.35 ± 11.44	14.37 ± 10.34	0.994	0.994	0.985	0.993
V_40_ (%)	7.96 ± 6.18	7.95 ± 5.97	8.01 ± 6.23	7.93 ± 7.27	0.997	0.989	0.994	0.963

All values are shown as the mean ± standard deviation

OAR, organ at risk; JT IMRT, jaw-tracking intensity-modulated radiation therapy; SJ IMRT, static jaw intensity-modulated radiation therapy; JT VMAT, jaw-tracking volumetric modulated arc therapy; FJ IMRT, fixed-jaw intensity-modulated radiation therapy; MLD, mean lung dose; V_x_, the relative volume of an OAR receives a dose of at least x Gy; D_max_, maximum dose; D_mean_, mean dose

Table 5 shows the TCP of PTV-G and PTV-C and the NTCP of the total lung and spinal cord. The TCP of PTV-G in the FJ IMRT plan was 65.48 ± 0.56, which was similar to that in the JT IMRT (65.45 ± 0.70), SJ IMRT (65.43 ± 0.72) and JT VMAT plans (65.51 ± 0.64), and there were no significant differences (*p* > 0.05). The NTCP of the total lung in the SJ IMRT, JT IMRT, JT VMAT and FJ IMRT plans were sequentially reduced, but no significant differences were detected between the JT VMAT and FJ IMRT plan. For NTCP_spinal_cord_, there were no significant differences between the FJ IMRT plan and the other three plans. The NTCP of the heart for all plans was lower than 0.1%; therefore, this study did not conduct a statistical analysis of this parameter.

**Table 5:** Comparisons of TCP and NTCP. All values are shown as the mean ± standard deviation

	JT IMRT	SJ IMRT	JT VMAT	FJ IMRT	ANOVA *p* value	Post hoc *p* value		
						FJ IMRT versus JT IMRT	FJ IMRT versus SJ IMRT	FJ IMRT versus JT VMAT
TCP_PTV-G_ (%)	65.45 ± 0.70	65.43 ± 0.72	65.51 ± 0.64	65.48 ± 0.56	0.969	0.908	0.756	0.862
TCP_PTV-C_ (%)	56.60 ± 2.53	56.58 ± 2.55	56.63 ± 2.46	56.66 ± 2.37	0.999	0.918	0.875	0.972
NTCP_total_lung_ (%)	5.04 ± 1.83	5.51 ± 2.06	4.55 ± 1.56	4.29 ± 1.41	0.021	0.044	0.004	0.537
NTCP_spinal_cord_ (%)	0.69 ± 0.23	0.73 ± 0.24	0.80 ± 0.34	0.70 ± 0.35	0.442	0.800	0.718	0.202

TCP, tumor control probability; NTCP, normal tissue complication probability; JT IMRT, jaw-tracking intensity-modulated radiation therapy; SJ IMRT, static jaw intensity-modulated radiation therapy; JT VMAT, jaw-tracking volumetric modulated arc therapy; FJ IMRT, fixed-jaw intensity-modulated radiation therapy

The correlations between length of PTV-C_inferior_ and the amount of difference in dosimetric parameters and NTCP of lungs in FJ IMRT plan relative to the other three plans are shown in Table 6. The length of PTV-C_inferior_ is moderately positively associated with ΔMLD_JT-FJ_, ΔV_5 JT-FJ_, ΔV_10 JT-FJ_, ΔV_13 JT-FJ_, ΔV_15 JT-FJ_, and ΔNTCP_JT-FJ_ showed. Similarly, the length of PTV-C_inferior_ and ΔMLD_SJ-FJ_, ΔV_5 SJ-FJ_, ΔV_10 SJ-FJ_, ΔV_13 SJ-FJ_, ΔV_15 SJ-FJ_, and ΔNTCP_SJ-FJ_ also showed moderately positive correlations. However, a weak positive correlation was found between the length of PTV-C_inferior_ and ΔMLD_VMAT-FJ_, ΔV_5 VMAT-FJ_, ΔV_10 VMAT-FJ_, ΔV_13 VMAT-FJ_ and ΔNTCP_VMAT-FJ_. No significant correlations were found between the length of PTV-C_inferior_ and other parameters. In summary, the presented results suggested that, with the increase of extension of the target below the upper boundary of lung tissue, the reduction of low-dose volume of the total lung was clearly larger for FJ IMRT respect to SJ IMRT and JT IMRT, but that there was a less clear advantage compared to JT VMAT.

**Table 6:** Correlation between length of PTV-C_inferior_ and the amount of difference in dosimetric parameters and NTCP of lungs in FJ IMRT plan relative to JT IMRT, SJ IMRT and JT VMAT plans

Reduction in FJ IMRT plan comparing with JT IMRT plan	Length of PTV-C_inferior_ (cm)		Reduction in FJ IMRT plan comparing with SJ IMRT plan	Length of PTV-C_inferior_ (cm)		Reduction in FJ IMRT plan comparing with JT VMAT plan	Length of PTV-C_inferior_ (cm)	
	Pearson’s rank			Pearson’s rank			Pearson’s rank	
	R	*P*		R	*P*		R	*P*
Total lung			Total lung			Total lung		
ΔMLD_JT-FJ_	0.736	< 0.001	ΔMLD_SJ-FJ_	0.745	< 0.001	ΔMLD_VMAT-FJ_	0.401	0.017
ΔV_5 JT-FJ_	0.707	0.037	ΔV_5 SJ-FJ_	0.797	0.018	ΔV_5 VMAT-FJ_	0.498	0.004
ΔV_10 JT-FJ_	0.691	0.009	ΔV_10 SJ-FJ_	0.697	0.005	ΔV_10 VMAT-FJ_	0.468	0.017
ΔV_13 JT-FJ_	0.659	0.005	ΔV_13 SJ-FJ_	0.602	< 0.001	ΔV_13 VMAT-FJ_	0.405	0.026
ΔV_15 JT-FJ_	0.523	0.001	ΔV_15 SJ-FJ_	0.673	< 0.001	ΔV_15 VMAT-FJ_	0.124	0.145
ΔV_20 JT-FJ_	0.026	0.133	ΔV_20 SJ-FJ_	0.308	0.072	ΔV_20 VMAT-FJ_	0.146	0.462
ΔV_30 JT-FJ_	0.108	0.536	ΔV_30 SJ-FJ_	0.085	0.628	ΔV_30 VMAT-FJ_	0.029	0.792
ΔNTCP_total_lung JT-FJ_	0.561	< 0.001	ΔNTCP_total_lung SJ-FJ_	0.618	< 0.001	ΔNTCP_total_lung VMAT-FJ_	0.419	0.038

PTV-C_inferior_, the part of the PTV-C below the upper boundary of lung tissue; NTCP, normal tissue complication probability; JT IMRT, jaw-tracking intensity-modulated radiation therapy; SJ IMRT, static jaw intensity-modulated radiation therapy; JT VMAT, jaw-tracking volumetric modulated arc therapy; FJ IMRT, fixed-jaw intensity-modulated radiation therapy; MLD, mean lung dose; V_x_, the relative volume of an OAR receives a dose of at least x Gy; ΔX_Y-FJ_, the difference in a dosimetric parameter (MLD, V_x_) or NTCP of the total lung between the Y plan and FJ IMRT plan

The total MUs and treatment time are summarized in Table 7. The total MUs and treatment time in the FJ IMRT plan were similar to those in the JT IMRT plan, but higher than those in the SJ IMRT plan or the JT VMAT plan. No statistically significant differences were observed in γ passing rates, which were on average ≥ 95% for the four techniques. Therefore, the JT VMAT plan was the one with the lowest number of MUs and the shortest duration.

**Table 7:** MUs, delivery time and γ passing rate

	JT IMRT	SJ IMRT	JT VMAT	FJ IMRT	ANOVA p value	Post hoc p value		
						FJ IMRT versus JT IMRT	FJ IMRT versus SJ IMRT	FJ IMRT versus JT VMAT
MUs	937 ± 194	713 ± 137	517 ± 59	888 ± 99	< 0.001	0.122	< 0.001	< 0.001
Delivery time (s)	522 ± 5.6	488.8 ± 45.2	263.7 ± 43.3	516.1 ± 54.7	< 0.001	0.610	0.021	< 0.001
γ passing rate (%)	96.3 ± 2.1	96.0 ± 1.5	95.8 ± 2.4	96.9 ± 1.7	0.456	0.891	0.769	0.335

All values are shown as the mean ± standard deviation

MU, Monitor unit; JT IMRT, jaw-tracking intensity-modulated radiation therapy; SJ IMRT, static jaw intensity-modulated radiation therapy; JT VMAT, jaw-tracking volumetric modulated arc therapy; FJ IMRT, fixed-jaw intensity-modulated radiation therapy

## Discussion

This study explored the potential dosimetric and biological benefits of the fixed-jaw IMRT technique for T-shaped esophageal cancer by comparing it with the jaw tracking IMRT, static jaw IMRT and jaw tracking VMAT techniques. The results showed that the FJ IMRT technique could minimize the volume of lung tissue receiving low doses while providing a target coverage comparable with the other techniques. Nevertheless, MUs were higher and delivery time was longer than with JT VMAT.

This study mainly focused on T-shaped esophageal cancer. This type of target shape is common among esophageal cancer patients treated in our institution. On average, nearly 600 esophageal cancer patients are treated in our center each year, of which nearly 200 patients present the T-shaped target volume with different lengths of the target below the upper boundary of lung tissue. The anatomy surrounding the target can vary greatly, and the tumors can be close to the spinal cord, lung and heart. Therefore, with the target extending longer below the upper boundary of lung tissue, it’s a major challenge to achieve a satisfactory target coverage whilst sparing the OARs.

The results showed that the dosimetric parameters of the FJ IMRT technique could meet the clinical requirements like the other three techniques; the D_2_, D_98_, conformity and homogeneity for the PTV-G and PTV-C with the FJ IMRT plan were not significantly different from those of the JT IMRT and SJ IMRT plans, and this was consistent with the study from Wang et al. [16] and Song et al. [18]. The results of this study also showed that the TCP of the FJ IMRT plan was similar to that of the JT IMRT, SJ IMRT and JT VMAT plans, and the absolute differences were small. As expected, our results indicated that compared with conventional techniques for T-shaped esophageal cancer, the FJ IMRT technique could also achieve sufficient target conformity and homogeneity.

A few investigators have reported the dosimetric advantages of VMAT with jaw tracking in head and neck, thorax, abdomen, and pelvis patients, due to a reduced transmitted radiation to the healthy tissue laying beneath the tracking jaws. [29–31]. Pokhrel et al. showed that VMAT with a jaw tracking plan exhibited superior OARs sparing compared to the no jaw tracking VMAT plan in the given complexity of a single-isocenter/two-lesion lung SBRT setting [29]. In a previous study, we demonstrated that compared to the IMRT plan with jaw tracking, the full arc VMAT plan with jaw tracking could reduce the V_20_, V_30_ and MLD of the total lung while increasing the V_5_, V_10_, V_13_, V_15_ of the total lung for upper thoracic esophageal cancer.

The partial arc VMAT plan with jaw tracking instead, significantly decreased the V_20_, V_30_ and MLD of the total lung while maintaining V_5_, V_10_, V_13_ and V_15_ similar to IMRT. This was why the partial arc VMAT technique with jaw tracking was chosen to conduct this study.

RP, which is closely correlated to the dose received by the lungs, is an important dose-limiting toxicity in esophageal cancer radiotherapy. Dose-volume parameters, such as V_5_, V_20_, V_30_ and MLD of the total lung, have also been reported to be correlated with the development of RP in thoracic radiotherapy [32–37]. Wang et al. also found that V_5_ was the only independent dosimetric factor associated with RP [37]. V_5_ and V_10_ were reported as the only factors significantly associated with grade ≥ 2 RP in esophageal cancer patients receiving chemoradiotherapy [38]. Schallenkamp et al. [35] reported that V_20_ and MLD were also important predictors of RP. Hernando et al. [39] found that unlike the dosimetry factors of V_30_ and MLD, NTCP alone was the single best predictor of RP. Therefore, reducing the lung volume to receive a lower dose has clinical significance, and it is necessary to reduce all doses to lung tissue as much as possible in esophageal cancer patients receiving external beam radiotherapy. In this study, we evaluated lung sparing in T-shaped esophageal cancer radiotherapy using the fixed-jaw IMRT technique. The results showed that the V_20_, MLD and low-dose lung volumes, including V_5_, V_10_, V_13_, and V_15_ of the total lung, were significantly reduced with the FJ IMRT plan compared to the JT/SJ IMRT plan. This finding was similar to the conclusion by Wang et al. [16]. This may be mainly resulting from the different beam arrangements, which reduces the dose to the lung tissue. The results of this study also showed that compared with JT VMAT plan, the FJ IMRT plan significantly reduced the V_5_, V_10_ and V_13_ of the total lung. Hernando et al. reported that the V_30_ of the total lung was an important predictor of RP [39], and a V_30_ of 18% was accompanied by a 6% RP rate compared with a 24% rate in patients with a V_30_ > 18%. Our results showed that the V_30_ of all plans was much lower than 18%.

We not only analyzed the dosimetric parameters but also calculated the TCP and NTCP values. The results showed that the NTCP of the total lung was the lowest in the FJ IMRT plan, but it was not significantly different from that in the JT VMAT plan. Thus, the dose reduction to the lung tissue achieved with FJ IMRT compared to the other approaches, may potentially translate into a reduced rate for RP in T-shaped esophageal cancer patients. Further comprehensive clinical studies and clinical evidence are necessary to rule out a decisive clinical advantage of FJ IMRT technique vs other standard techniques.

The results showed that the length of PTV-C_inferior_ exhibited a moderately positive correlation with each reduction (MLD, V_5_, V_10_, V_13_, V_15_ and NTCP) in the FJ IMRT plan relative to the JT IMRT or SJ IMRT plan, respectively. However, the length of PTV-C_inferior_ exhibited a weakly positive correlation with each reduction (MLD, V_5_, V_10_, V_13_ and NTCP) in the FJ IMRT plan relative to the JT VMAT plan. This finding thus suggests that as the length of PTV-C_inferior_ increases, the FJ IMRT technique could more effectively reduce the low-dose volume lung tissue than the JT IMRT, SJ IMRT and JT VMAT techniques. The length of PTV-C_inferior_ did not exhibit any correlation with the reduction in V_20_ and V_30_ in the FJ IMRT plan relative to the other three plans, which is probably caused by the small difference in the lungs’ volume in the immediate proximity of the MLC apertures receiving the higher dose levels of 20 or 30 Gy.

In clinical practice, when the PTV-C_inferior_ in T-shaped esophageal cancer extends very long, it will be difficult to meet the clinical requirements for the lungs’ volume receiving low doses. In such cases, the FJ IMRT technique proposed in this study can be advantageous.

As shown in Table 6, the delivery time and MUs of FJ IMRT plan were the highest in four plans. For some patients, it is difficult to stay in the same position for a long time, and delivery time is a factor to be considered. From this point of view, this may be another inadequacy of the FJ IMRT technique. On the other hand, the dosimetric results achieved with the FJ-IMRT technique for the lungs’ volume exposed to low-doses, suggest that the increase in the contribution from scattered radiation associated with a higher number of MUs was minor compared to the dose reduction in radiation leakage and transmission gained by locking the jaws.

The aim of dose verification in this study is to check that the calculated dose distributions of the four techniques could be reliably delivered, not to compare the difference in γ passing rates between them. The result showed that all the γ passing rates of four plans were above 95%, which indicated that the dose distribution of four plans could meet the clinical requirement.

Our results showed that the effect of the FJ IMRT plan was slightly better than that of the JT VMAT plan in lung sparing. However, there are many factors to be considered in the selection of planning and treatment techniques, including treatment time. The treatment time of the FJ IMRT plan was almost twice that of the JT VMAT plan. Therefore, when considering patient discomfort or large respiratory movements, the JT VMAT plan might be preferred over the FJ-IMRT plan for medical centers with a VMAT accelerator, despite the increased lung exposure. For many medical centers in developing countries without VMAT accelerators, the FJ IMRT approach is also quite a good choice for T-shaped esophageal cancer radiotherapy.

## Conclusion

In summary, comparing with the jaw tracking IMRT, static jaw IMRT and jaw tracking VMAT techniques, the fixed-jaw IMRT technique could provide comparable target coverage and the better lung sparing at low-dose region for T-shaped esophageal cancer radiotherapy, especially for the patient with long target extending below the upper boundary of lung tissue. In cases where time is not an important factor or where VMAT is not available, the fixed-jaw IMRT technique is a great possibility for planning esophageal cancer patients with reduced lung exposure and therefore potentially reduced risk of developing radiation pneumonitis.

## Data Availability

All data included in this study are available upon request by contact with the corresponding author.

## Abbreviations

RP: Radiation pneumonitis
IMRT: Intensity-modulated radiation therapy
VMAT: Volumetric modulated arc therapy
MLC: Multi-leaf collimator
PTV: Planning target volume
OAR: Organ at risk
MU: Monitor unit
GTV: Gross tumor volume
CTV: Clinical target volume
JT: Jaw tracking
SJ: Static jaw
FJ: Fixed-jaw
MLD: Mean lung dose
PTV-C_superior_: The part of the PTV-C above the upper boundary of lung tissue
PTV-C_inferior_: The part of the PTV-C below the upper boundary of lung tissue
DVH: Dose-volume histogram
CI: Conformity index
HI: Homogeneity index
TCP: Tumor control probability
NTCP: Normal tissue complication probability
EUD: Equivalent uniform dose
